# Describing well-being in the US by race/ethnicity, education level, and urbanicity: A cross-sectional study using the Gallup National Health and Well Being Index

**DOI:** 10.1371/journal.pone.0353745

**Published:** 2026-09-16

**Authors:** Brita Roy, Jeph Herrin, Erica S. Spatz, Kiarri N. Kershaw, Dan Witters, Harlan M. Krumholz, Carley Riley

**Affiliations:** 1 Section for Health Equity, Department of Population Health, New York University Grossman School of Medicine, New York, New York, United States of America; 2 Division of General Internal Medicine and Clinical Innovation, Department of Medicine, New York University Grossman School of Medicine, New York, New York, United States of America; 3 The Nova Institute, Baltimore, Maryland, United States of America; 4 Section of Cardiovascular Medicine, Department of Medicine, Yale School of Medicine, New Haven, Connecticut, United States of America; 5 Center for Outcomes Research and Evaluation, Yale-New Haven Health, New Haven, Connecticut, United States of America; 6 Department of Preventive Medicine, Northwestern University Feinberg School of Medicine, Chicago, Illinois, United States of America; 7 Gallup, Inc., Omaha, Nebraska, United States of America; 8 Department of Health Policy and Management, Yale School of Public Health, New Haven, Connecticut, United States of America; 9 Department of Clinical Pediatrics, University of Cincinnati College of Medicine, Cincinnati, Ohio, United States of America; 10 Division of Critical Care, Cincinnati Children’s Hospital Medical Center, Cincinnati, Ohio, United States of America; De Montfort University, UNITED KINGDOM OF GREAT BRITAIN AND NORTHERN IRELAND

## Abstract

For a more comprehensive understanding of health disparities, consideration must be given not only to morbidity and mortality outcomes but also to more holistic health-related constructs, such as well-being. We assessed differences in well-being across sociodemographic subgroups in the United States (US). We used data from the Gallup National Health and Well-being Index (2014–2016, n = 530,920) to assess independent cross-sectional associations of self-reported race, ethnicity, educational attainment, and rural residence with measures of well-being for non-institutionalized residents from 50 US states and the District of Columbia. Our outcome measures are thriving, calculated from self-reported current and future life evaluation, and the composite Well-Being Index (WBI), calculated from 38 self-reported metrics spanning 5 domains of well-being (Career, Community, Physical, Financial, Social). We summarized these measures for race-education-urban subgroups and tested for interactions among these three factors, before and after adjustment for year of survey completion, age, sex, marital status, household size, and income. Among our sample, 55% were thriving. After adjustment, higher levels of education were associated with increased odds of thriving: compared with those with no high school degree, we found higher odds of thriving for respondents reporting completion of high school/technical school/some college (OR=1.61(1.57,1.66)), college (OR=2.77(2.69,2.85)), and postgraduate (OR=3.38 (3.29,3.49)) education. Compared with White respondents, Black (OR (95% CI)=1.15 (1.12,1.17)) and Hispanic (OR=1.26 (1.23,1.29)) respondents had higher odds of thriving; results were similar for WBI, except Asian respondents had also higher WBI after adjustment for all covariates (OR=2.54 (2.28,2.80)). Across race-education-urban subgroups, non-White respondents with a college degree living in urban and rural areas had the highest odds of thriving; White respondents with no college degree living in urban areas had the lowest odds of thriving (p < 0.001 for each), after adjustment. These findings raise questions about how well-being is mediated given the known worse clinical health measures among minoritized groups.

## Introduction

Well-being is a holistic, positively framed, person-centered assessment of health and quality of life that describes “how people think, feel, and function—at a personal and social level—and how they evaluate their lives as a whole” [1]. It incorporates multiple important aspects of one’s life, including perceived health, social support, connection to and contribution to the community, opportunity for growth and advancement, liking what you do each day and having a sense of purpose in life [2,3]. It also can incorporate resilience.

At the population level, there is heterogeneity in well-being by where people live. Within the US, the percent population thriving by counties ranges from 38.3% to 69.6% [4]. The sociodemographic composition of these counties may be associated with its residents’ reported well-being. Prior studies of this topic are small and have not been able to account for the complex relationships between race, ethnicity, education levels, and rural/urban living contexts that exist due to the effects of longstanding structural racism and classism, and therefore, do not exist in isolation in the US. As such, evaluating associations of these sociodemographic characteristics and their potential interactions with respect to well-being may offer new insights on the health of populations.

Accordingly, this study aims to assess differences in well-being and its elements across various sociodemographic groups and to explore whether interactions among these sociodemographic characteristics exists. The Gallup National Health and Well-Being Index is an ideal data source, as the largest and most comprehensive assessment of well-being in the US, to address these aims. The survey includes a measure of overall thriving (reflecting current and anticipated high life evaluation) in addition to a multi-dimensional assessment of well-being and its elements, including Career (e.g., liking what you do), Community (e.g., satisfaction with living in one’s community), Physical (e.g., physical health and functioning), Financial (e.g., financial security), and Social (e.g., quality of social relationships). [5] In this study, we use survey data from more than one-half million Americans to describe thriving, well-being, and its elements among different sociodemographic subgroups, highlighting differences across historically disadvantaged groups defined by race/ethnicity, education, and urbanicity, as well as combinations of these characteristics.

## Materials and methods

### Data source

We used data from the Gallup National Health and Well-Being Index, previously known as the Gallup-Sharecare Well-Being Index, collected January 1^st^, 2014 to December 31^st^, 2016, to conduct this cross-sectional study assessing differences in levels of well-being associated with self-reported sociodemographic characteristics. Trained Gallup interviewers performed a computer-assisted telephone interview with a unique daily sample of 500–1000 adults over 18 years of age, each of 350 days per year. A structured, dual-frame sampling design (including random-digit-dialing) was used to recruit respondents from all 50 states and the District of Columbia. The 16-minute survey was administered in both English and Spanish, using both landlines and cellular phones. The resulting sample is estimated to represent more than 95% of the US population. The Yale University Human Subjects Committee approved this research and waived the need for consent.

### Well-being measures

We include two primary measures of well-being from the survey in this study: thriving and a multi-dimensional well-being composite score (WBI composite). Cantril’s Self-Anchoring Striving Scale [6] is a well-validated measure of self-reported overall life evaluation that was used to calculate whether respondents were thriving. These items ask respondents to rate on a ladder scale from 0 to 10 their overall current life satisfaction as well as their anticipated life satisfaction in five years. Based on their responses, participants are categorized as thriving if their current life satisfaction is rated a seven or higher and their anticipated life satisfaction is rated an eight or higher [7].

To develop the WBI composite, survey items that aligned with prior research on well-being were compiled by experts in the field [8–10]. Based on the existing literature, items were selected so that the survey would include eudaimonic well-being (i.e., an individual’s judgments about the meaning and purpose in one’s life) and hedonic well-being (i.e., people’s feelings and their thoughts about their lives) [11]. The survey therefore includes items assessing meaning and purpose in life, daily emotional experience, and a wide variety of evaluative items, such as satisfaction with standard of living, community, work, relationships, and personal health. Data from a large, representative national sample was then used to perform factor analysis to determine the final set of questions. Criterion validity of geographically aggregated data was established by examining correlations with health and socioeconomic indicators [12]. Principal component and confirmatory factor analyses were then used to create an instrument valid for measuring individual well-being. The well-being measure has good reliability, internal and external validity [5].

From 2014–2016, the composite WBI was comprised of 38 scored metrics derived from five elements: Career, Community, Physical, Financial, and Social. The Career element assesses how much respondents like what they do, and includes the usage of natural talents and strengths, learning and growing, and setting and reaching goals. The Community element examines satisfaction with living in the community and factors that inform this condition including impactful volunteerism and personal safety. The Physical element includes measures of comorbid conditions, health behaviors, and physical functioning. The Financial element assesses managing one’s wealth and living within one’s means to build financial security. The Social element examines the presence of positive social relationships. As with the WBI composite score, each element is computed on a rebased scale from 0 to 100.

### Sociodemographic characteristics

Participants were also asked about their sociodemographic characteristics, including age, sex, race, ethnicity, education, marital status, income, number of adults and children in the household, and zip code of residence. We grouped age into the following non-overlapping categories: <=25, 26–35, 36–45, 46–55, 56–65, 66–75, 76–85, and >=86 years. Sex was recorded as being either male or female. We categorized self-identified primary race and ethnicity into five mutually exclusive groups: Asian, Hispanic, non-Hispanic White (White), non-Hispanic Black (Black), or other, which included those who identified as American Indian, Native Hawaiian, or Pacific Islander. We categorized the highest level of education achieved as less than high school, high school/technologic degree/some college, college, and post-graduate degree. Marital status was recorded as being single/never been married, married, separated, divorced, widowed, or domestic partnership. We categorized annual household income into the following non-overlapping categories: < $24,000, $24,000 - $47,999, $48,000 - $119,999, >=$120,000, and refused. Number of adults in the household was recorded as 1, 2, 3, 4, or >=5. Number of children in the household was recorded as 0, 1, 2, 3, 4, or >=5. We used the 2010 Census Urban and Rural Classification and Urban Area Criteria to categorize participants’ reported residential zip code into one of three groups: rural area, small urban area, and large urban area [13].

We created categories that combined the three characteristics of race/ethnicity, education, and urban/rural residence designation (race-education-urban subgroups) to assess whether the interaction of these sociodemographic characteristics was associated with thriving, the WBI composite and its elements. For these subgroups, we recategorized each sociodemographic characteristic into a binary variable. We stratified race/ethnicity by whether a participant identified as White, because White was the largest racial group in the sample and because the other racial groups experience minoritization. We stratified education by whether a college degree or higher was achieved because prior literature reports higher well-being among college-educated individuals. We collapsed urban designation into either living inside an urbanized area or an urban cluster because we hypothesized these groups would be more similar than rural areas.

### Analysis

We first summarized thriving (percent) and WBI scores (mean and standard deviation) for each sociodemographic characteristic: age, sex, race/ethnicity, education, urban/rural, income, and marital status. We used logistic and linear regression to test for differences in thriving status and WBI across characteristics, respectively, reporting overall Wald P-values for each characteristic. We then used a sequential approach to identify the independent associations of these sociodemographic characteristics with the measures of well-being. We first examined bivariate associations between race/ethnicity, education, income, urban/rural categories with thriving and the WBI using logistic or linear models to test for differences among groups. Retaining all significant characteristics, we used singular decomposition to identify collinear variables and exclude those that were redundant [14]. We entered the retained characteristics in a series of sequential multivariable models to assess independent associations of race/ethnicity, education, and urban/rural designation with thriving and WBI composite and its elements: the base model (model 1) was adjusted for race/ethnicity, education, and/or urbanicity, accordingly; model 2 further adjusted model 1 for age and sex; model 3 further adjusted model 2 for marital status and household size; and model 4 further adjusted model 3 for income. Finally, we expanded the sequential multivariable models to include interactions for race/education/urbanicity. To avoid excessive hypothesis testing and spurious findings, we report confidence intervals for all effects, and P-values only for overall tests that all effects for given categorical characteristics are null.

All models accounted for correlation of measurements within counties by incorporating random intercepts across counties. Missing values were accounted for using multiple imputation, with 5 imputation sets. Level of statistical significance was set at ɑ = 0.05. All analyses were performed using Stata version 16.1 (StataCorp, College Station, TX).

## Results

We included data from 530,920 participants of the Gallup National Health and Well-Being Index (Table 1).

**Table 1:** Sociodemographic characteristics of our study sample and associated well-being measures (N = 530,920).

Socio-demographic Characteristic	n (%)	WBI (mean (SD))	P-value	% Thriving	P-value
**Full Sample**	530,920 (100)	62.9 (14.4)		54.8	
**Age (categories)**			<0.001		<0.001
<=25	48,251 (9.1)	62.1 (13.8)		60.2	
26-35	60,182 (11.3)	60.9 (14.1)		62.3	
36-45	62,341 (11.7)	60.8 (14.2)		61.6	
46-55	86,152 (16.2)	60.4 (14.4)		55.5	
56-65	108,221 (20.4)	61.9 (14.7)		50.5	
66-75	96,780 (18.2)	65.9 (14.0)		47.6	
76-85	48,077 (9.1)	67.8 (13.0)		39.2	
86+	20,916 (3.9)	67.6 (14.4)		38.7	
**Sex**			<0.001		<0.001
Male	266,990 (50.3)	62.1 (14.1)		52.6	
Female	263,929 (49.7)	63.7 (14.6)		53.1	
**Race/ethnicity**			<0.001		<0.001
White	396,959 (74.8)	63.1 (14.3)		52.7	
Other	8180 (1.5)	61.1 (15.6)		48.0	
Black	47,403 (8.9)	60.6 (14.8)		52.8	
Asian	11,597 (2.2)	64.7 (12.5)		61.2	
Hispanic	50,171 (9.4)	63.3 (14.0)		53.8	
**Education**			<0.001		<0.001
Less than HS	31,103 (5.9)	60.0 (15.5)		34.5	
HS/Tech/Some College	276,047 (52.0)	61.5 (14.9)		47.0	
College	115,969 (21.8)	64.1 (13.4)		61.2	
Postgrad	99,534 (18.7)	66.2 (12.8)		65.6	
**Urbanicity**			<0.001		<0.001
Rural	55,844 (10.5)	62.9 (14.6)		47.6	
SUA	90,348 (17.0)	62.6 (14.7)		48.8	
LUA	369,207 (69.5)	63.0 (14.3)		54.6	
**Income**			<0.001		<0.001
< 24k	82,170 (15.5)	56.6 (16.1)		33.9	
24k-47.9k	100,954 (19.0)	60.7 (14.8)		43.4	
48k-119.9k	170,219 (32.1)	64.1 (13.2)		58.7	
120k+	81,029 (15.3)	66.7 (12.0)		73.3	
**Marital Status**			<0.001		<0.001
Single/Never married	101,733 (19.2)	59.6 (14.7)		52.0	
Married	276,436 (52.1)	65.3 (13.2)		58.7	
Separated	9899 (1.9)	55.1 (15.6)		39.2	
Divorced	57,193 (10.8)	57.9 (15.6)		42.7	
Widowed	52,962 (10.0)	64.8 (14.5)		37.3	
Domestic partnership	25,345 (4.8)	60.9 (14.5)		53.6	

Abbreviations: SUA=small urban area; LUA=large urban area.

Participants were 50% female and 75% White. Fifty-eight percent of participants had less than a college degree and 35% had lower than $48,000 annual household income. Eleven percent of participants lived in areas designated as rural. Fifty-five percent of participants were categorized as thriving. Characteristics associated with thriving were younger age, female sex, Asian race/ethnicity, higher educational attainment, having higher annual income, urbanicity, and being married. The mean WBI composite score among all participants was 62.9 (SD 14.4). Characteristics associated with higher WBI composite included older age, female sex, Asian race/ethnicity, higher educational attainment, having higher annual household income, living in rural area, and living with a spouse/significant other or being widowed.

We found differences in odds of thriving and WBI composite scores among education, urban/rural and race/ethnic groups, in our base and fully adjusted models (Table 2). Singular decomposition analysis found no collinearity, so all bivariate factors were carried forward to multivariable models.

**Table 2:** Associations of sociodemographic characteristics with percent thriving and WBI composite score. Model 1: Base model: Race/ethnicity models adjusted for education and urbanicity; Education models adjusted for race/ethnicity and urbanicity; Urbanicity models adjusted for race/ethnicity and education. Model 2: Adjusted base model for age and sex. Model 3: Model 2 further adjusted for marital status and household size. Model 4: Model 3 further adjusted for income.

Sociodemographic Subgroup	Thriving (OR (95% CI))				WBI composite (β (95% CI))			
	Model 1	Model 2	Model 3	Model 4	Model 1	Model 2	Model 3	Model 4
**Race/ethnicity**								
White	ref	ref	ref	ref	ref	ref	ref	ref
Other	0.95 (0.90,0.99)	0.85 (0.81,0.89)	0.88 (0.84,0.93)	0.95 (0.90,0.99)	−1.31 (−1.63, − 0.98)	−0.30 (−0.62,0.01)	0.16 (−0.15,0.47)	0.71 (0.40,1.01)
Black	1.08 (1.05,1.10)	0.95 (0.93,0.97)	1.05 (1.03,1.07)	1.15 (1.12,1.17)	−1.58 (−1.72, − 1.43)	−0.68 (−0.82, − 0.54)	0.37 (0.23,0.51)	1.04 (0.90 , 1.17)
Asian	1.13 (1.08,1.17)	0.89 (0.85,0.93)	0.90 (0.86,0.94)	0.92 (0.88,0.95)	0.79 (0.52,1.07)	2.24 (1.97,2.51)	2.31 (2.04,2.58)	2.54 (2.28,2.80)
Hispanic	1.35 (1.32,1.38)	1.09 (1.06,1.11)	1.11 (1.08,1.13)	1.26 (1.23,1.29)	1.34 (1.19,1.49)	2.86 (2.71,3.01)	3.05 (2.90,3.20)	4.02 (3.87,4.16)
Overall p-value	<0.001	<0.001	<0.001	<0.001	<0.001	<0.001	<0.001	<0.001
**Education**								
< HS	ref	ref	ref	ref	ref	ref	ref	ref
HS/Tech/ Some college	1.61 (1.57,1.66)	1.58 (1.54,1.62)	1.51 (1.47,1.55)	1.27 (1.24,1.31)	1.77 (1.60,1.95)	2.11 (1.94,2.28)	1.58 (1.41,1.75)	0.01 (−0.16,0.17)
College	2.77 (2.69,2.85)	2.66 (2.58,2.73)	2.44 (2.37,2.52)	1.77 (1.72,1.83)	4.24 (4.05,4.43)	5.23 (5.05,5.42)	4.17 (3.98,4.35)	1.41 (1.22,1.59)
Postgrad	3.38 (3.29,3.49)	3.48 (3.38,3.59)	3.16 (3.07,3.26)	2.14 (2.07,2.21)	6.39 (6.19,6.58)	6.85 (6.66,7.04)	5.59 (5.40,5.78)	2.35 (2.16,2.54)
p-trend	<0.001	<0.001	<0.001	<0.001	<0.001	<0.001	<0.001	<0.001
**Urbanicity**								
Rural	ref	ref	ref	ref	ref	ref	ref	ref
SUA	1.02 (0.99,1.04)	1.01 (0.99,1.03)	1.02 (1.00,1.04)	1.01 (0.99,1.03)	−0.44 (−0.59, − 0.28)	−0.48 (−0.63, − 0.32)	−0.32 (−0.47, − 0.17)	−0.39 (−0.54, − 0.24)
LUA	1.12 (1.09,1.14)	1.09 (1.07,1.11)	1.12 (1.10,1.15)	1.05 (1.03,1.07)	−0.55 (−0.71, − 0.39)	−0.50 (−0.65, − 0.35)	−0.20 (−0.34, − 0.05)	−0.62 (−0.76, − 0.47)
p-trend	<0.001	<0.001	<0.001	<0.001	<0.001	<0.001	<0.001	<0.001

Abbreviations: HS=high school; Tech=technical school; SUA=small urban area; LUA=large urban area.

### Education

Higher levels of educational achievement were associated with higher odds of thriving and higher WBI scores in a graded fashion (p-trends <0.001). These relationships were slightly attenuated but persisted after adjustment for all covariates (p-trends <0.001). Each additional level of education was associated with higher odds of thriving and higher WBI scores (all 95% CIs > 1 for thriving and > 0 for WBI) with one exception: those with a high-school diploma, technical degree, or some college had similar WBI scores to those without a high school diploma (b = 0.01 (−0.16,0.17)).

### Rural/urban

Compared with living in rural areas, living in large urban areas was associated with higher odds of thriving in our base model and after adjustment for all covariates (overall p-values <0.001; Table 2). However, those living in large urban areas had lower WBI composite scores compared with those living in rural areas, even in fully adjusted models (b = −0.62(−0.76,-0.47)).

### Race/ethnicity

Odds of thriving and WBI composite scores differed among race/ethnic subgroups (overall p-values <0.001 in all models), and our sequentially adjusted models revealed differential effects of confounding by other sociodemographic covariates on these relationships. Comparing Asian respondents with White respondents, Asian respondents had higher odds of thriving in Model 1 (OR (95% CI)=1.13 (1.08,1.17)), but after adjustment for all covariates they had lower odds of thriving (OR=0.92 (0.88,0.95)). For the WBI composite, compared with White respondents, Asian respondents had similar WBI scores (b = 0.79 (0.52,1.07)), but after adjustment for age, sex, and all other covariates, Asian respondents had higher WBI (b = 2.54 (2.28,2.80)). Compared with White respondents, Hispanic respondents had higher odds of thriving in all models (all 95% CIs above 1). Negative confounding with WBI scores was observed among this subgroup as well: compared with White respondents, Hispanic respondents had higher WBI scores in Model 1 (b = 1.34 (1.19,1.49)), and the association was stronger after adjustment for all covariates (b = 4.02 (3.87,4.16)).

When comparing Black respondents with White respondents, Black respondents had higher odds of thriving in the base model (OR=1.08 (1.05,1.10)) but lower odds of thriving in Model 2, after adjusting for age, sex, education, and urbanicity (Table 2). However, after further adjustment for marital status, household size, and income, Black respondents had higher odds of thriving (OR=1.15 (1.12,1.17)) (Table 2, Model 4). Similarly, the direction of associations with the WBI composite changed with sequential adjustment for sociodemographic covariates. In our base model, Black respondents had lower WBI scores than White respondents (b = −1.58 (−1.72,-1.43)). After adjustment for age and sex, Black respondents still had lower WBI scores than White respondents (b = −0.68 (−0.82,-0.54)), but after further adjustment for marital status, household size, and income, a higher relative association emerged between Black race and the WBI composite score (b = 1.04 (0.90–1.17)).

To further elucidate these changes in the directionality of the relative relationships between Black and White race and WBI composite across our sequential models, we assessed the effects of adjusting for each covariate associated with Black race separately. Black race was associated with younger age, having more children in the home, and lower income among the sample. When adjusting for each of these covariates separately, no single covariate changed the direction of the relative relationship with WBI composite. However, the combination of adjusting for age and income changed the direction of the relative relationship with WBI among Black respondents compared with White respondents, with further adjustment for marital status and household size having little additional impact on this relationship.

### WBI elements

Relative associations between sociodemographic subgroup categories and WBI element scores varied (Table 3).

**Table 3:** Associations of sociodemographic characteristics with WBI elements, reporting the base model and models further adjusted for all covariates, including year of survey completion, age, sex, marital status, income, and household size.

Sociodemographic Subgroup	Career β (SE)		Community β (SE)		Physical β (SE)		Financial β (SE)		Social β (SE)	
	Base	Adjusted	Base	Adjusted	Base	Adjusted	Base	Adjusted	Base	Adjusted
**Race/ethnicity**										
White	ref	ref	ref	ref	ref	ref	ref	ref	ref	ref
Other	−0.64 (−1.08, − 0.20)	1.96 (1.54,2.39)	−2.70 (−3.16, − 2.24)	−0.14 (−0.57,0.30)	−1.37 (−1.74, − 1.01)	0.67 (0.31,1.03)	−7.81 (−8.36, − 7.26)	−3.02 (−3.54, − 2.50)	−1.66 (−2.14, − 1.19)	1.51 (1.06,1.95)
Black	−0.85 (−1.04, − 0.66)	2.38 (2.19,2.57)	−4.17 (−4.37, − 3.98)	−0.87 (−1.06, − 0.67)	−0.82 (−0.98, − 0.65)	1.53 (1.37,1.70)	−9.88 (−10.12, − 9.64)	−4.44 (−4.68, − 4.20)	−1.98 (−2.19, − 1.77)	2.33 (2.13,2.54)
Asian	−0.36 (−0.73,0.00)	0.99 (0.63,1.36)	−0.11 (−0.49,0.26)	2.46 (2.09,2.83)	3.18 (2.87,3.49)	3.44 (3.13,3.75)	−0.20 (−0.67,0.26)	3.47 (3.02,3.92)	0.13 (−0.26,0.52)	1.47 (1.08,1.86)
Hispanic	1.96 (1.77,2.15)	5.33 (5.14,5.53)	0.41 (0.22,0.61)	4.29 (4.09,4.50)	1.05 (0.89,1.21)	3.89 (3.72,4.06)	−8.10 (−8.34, − 7.86)	−0.13 (−0.38,0.11)	−0.89 (−1.09, − 0.69)	3.43 (3.22,3.64)
P-value	<0.001	<0.001	<0.001	<0.001	<0.001	<0.001	<0.001	<0.001	<0.001	<0.001
**Education**										
< HS	ref	ref	ref	ref	ref	ref	ref	ref	ref	ref
HS/Tech/ Some College	−0.50 (−0.73, − 0.28)	−1.39 (−1.62, − 1.15)	−1.61 (−1.84, − 1.38)	−2.34 (−2.58, − 2.10)	0.96 (0.77,1.15)	0.22 (0.02,0.41)	5.86 (5.57,6.15)	4.37 (4.08,4.65)	2.03 (1.79,2.28)	0.08 (−0.17,0.33)
College	0.54 (0.30,0.78)	−1.19 (−1.44, − 0.93)	0.01 (−0.24,0.26)	−1.60 (−1.86, − 1.34)	3.81 (3.61,4.02)	2.59 (2.37,2.81)	10.18 (9.87,10.49)	8.90 (8.59,9.21)	4.85 (4.59,5.11)	1.70 (1.43,1.98)
Postgrad	3.52 (3.27,3.77)	0.47 (0.20,0.73)	2.15 (1.90,2.40)	−0.99 (−1.26, − 0.72)	5.33 (5.12,5.53)	3.32 (3.09,3.54)	14.89 (14.57,15.20)	11.67 (11.35,11.99)	6.68 (6.42,6.95)	2.18 (1.89,2.46)
P-trend	<0.001	<0.001	<0.001	<0.001	<0.001	<0.001	<0.001	<0.001	<0.001	<0.001
**Urbanicity**										
Rural	ref	ref	ref	ref	ref	ref	ref	ref	ref	ref
SUA	−0.01 (−0.22,0.20)	−0.12 (−0.33,0.08)	−2.29 (−2.52, − 2.07)	−2.30 (−2.51, − 2.08)	0.23 (0.04,0.41)	0.02 (−0.15,0.19)	0.13 (−0.15,0.41)	0.34 (0.09,0.59)	0.53 (0.30,0.75)	0.56 (0.35,0.77)
LUA	−0.19 (−0.39,0.02)	−0.76 (−0.95, − 0.57)	−3.52 (−3.76, − 3.28)	−3.60 (−3.82, − 3.37)	1.04 (0.86,1.23)	0.34 (0.17,0.51)	0.66 (0.38,0.94)	1.16 (0.92,1.40)	1.08 (0.87,1.29)	0.67 (0.48,0.87)
P-trend	0.079	<0.001	<0.001	<0.001	<0.001	<0.001	<0.001	<0.001	<0.001	<0.001

Abbreviations: HS = high school; Tech = technical school; SUA = small urban area; LUA = large urban area.

In the base models, non-White groups generally fared poorly in the Financial element compared with Whites (Black: −9.88(−10.12,-9.64); Asian: −0.20(−0.67,0.26); Hispanic: −8.10(−8.34,-7.86); Other: −7.81(−8.36,-7.26)), but these differences were attenuated after adjusting for income and other covariates. Compared with White respondents, Asian respondents on average scored 3.4 points higher in the Physical element (95%CI: 3.13,3.75) and 3.5 points higher in the Financial element (95%CI: 3.02,3.92), after adjusting for all covariates; Hispanic respondents scored a mean 5.3 points higher on the Career element (95%CI: 5.14,5.53) and 4.3 points higher on the Community element (95%CI: 4.09,4.50) in fully adjusted models; and Black respondents scored a mean 2.4 points higher on the Career element (95%CI: 2.19,2.57) and 2.3 points higher on the Social element (95%CI: 2.13,2.54). Generally, higher levels of education were associated with higher scores in all WBI elements with the exception of the Community element, after adjustment for income and other covariates. Relationships among element scores and categories of urbanicity were mixed, with lower Career and Community element scores and higher Physical, Financial, and Social element scores among large urban areas compared with rural areas.

### Race-education-urban intersectional subgroups

Finally, we observed differences in thriving as well as in the WBI and its elements among race-education-urban subgroups across sequentially adjusted models, with overall test of an interaction having P < 0.001 in all models (Tables 4 and 5).

**Table 4:** Associations of sociodemographic intersectional groups with percent thriving and WBI composite. Model 1: Unadjusted associations with race/ethnicity, education, and urbanicity. Model 2: Adjusted for age and sex. Model 3: Model 2 further adjusted for marital status and household size. Model 4: Model 3 further adjusted for income.

Intersectional Subgroup	N (%)	Thriving (OR (SE))				WBI composite (β (SE))			
	530920 (100)	Model 1	Model 2	Model 3	Model 4	Model 1	Model 2	Model 3	Model 4
<College/NW/R	5430 (1.1)	ref	ref	ref	ref	ref	ref	ref	ref
<College/NW/U	78158 (15.3)	1.09 (1.03,1.16)	1.03 (0.97,1.10)	1.04 (0.98,1.11)	1.03 (0.97,1.10)	0.08 (−0.36,0.52)	0.20 (−0.24,0.64)	0.30 (−0.14,0.75)	0.27 (−0.16,0.70)
<College/W/R	34706 (6.8)	0.89 (0.83,0.95)	1.01 (0.95,1.08)	0.94 (0.88,1.00)	0.84 (0.79,0.90)	0.47 (0.02,0.92)	−0.55 (−1.00, − 0.10)	−1.38 (−1.83, − 0.93)	−2.20 (−2.64, − 1.76)
<College/W/U	185394 (36.2)	0.96 (0.90,1.02)	1.08 (1.02,1.15)	1.03 (0.97,1.10)	0.89 (0.84,0.95)	0.06 (−0.36,0.49)	−1.06 (−1.49, − 0.64)	−1.62 (−2.04, − 1.19)	−2.74 (−3.16, − 2.33)
College + /NW/R	1424 (0.3)	1.98 (1.74,2.26)	2.05 (1.79,2.34)	1.96 (1.71,2.24)	1.54 (1.34,1.76)	3.92 (2.98,4.87)	4.03 (3.09,4.96)	3.30 (2.41,4.19)	1.27 (0.41,2.14)
College + /NW/U	38414 (7.5)	2.05 (1.92,2.18)	2.01 (1.88,2.14)	1.92 (1.80,2.05)	1.46 (1.37,1.56)	3.01 (2.56,3.46)	3.40 (2.95,3.85)	2.75 (2.31,3.20)	0.63 (0.20,1.07)
College + /W/R	14284 (2.8)	1.73 (1.62,1.86)	2.01 (1.87,2.15)	1.79 (1.67,1.92)	1.35 (1.26,1.45)	4.42 (3.94,4.90)	3.61 (3.13,4.09)	2.22 (1.75,2.69)	−0.03 (−0.48,0.43)
College + /W/U	157589 (30.8)	1.88 (1.77,1.99)	2.14 (2.01,2.27)	1.94 (1.82,2.06)	1.39 (1.30,1.48)	3.75 (3.32,4.18)	3.12 (2.69,3.55)	1.90 (1.47,2.33)	−0.63 (−1.05, − 0.21)
p-value*		<0.001	<0.001	<0.001	<0.001	<0.001	<0.001	<0.001	<0.001

Abbreviations: NW = non-White; W = White; R = rural; U = small or large urban area.

* P-value is for an overall Wald test that all interaction terms are jointly 0.

**Table 5:** Associations of sociodemographic intersectional subgroups with WBI elements, adjusted for year of survey completion, age, sex, marital status, income, and household size.

Intersectional Subgroup	Career β (SE)	Community β (SE)	Physical β (SE)	Financial β (SE)	Social β (SE)
<College/NonWhite/Rural	ref	ref	ref	ref	ref
<College/NonWhite/Urban	0.46 (−0.10,1.02)	−1.87 (−2.45, − 1.28)	0.85 (0.36,1.35)	0.84 (0.07,1.60)	0.81 (0.21,1.42)
<College/White/Rural	−3.54 (−4.12, − 2.95)	−1.21 (−1.80, − 0.61)	−2.30 (−2.80, − 1.81)	2.47 (1.67,3.26)	−2.78 (−3.41, − 2.16)
<College/White/Urban	−3.90 (−4.45, − 3.35)	−4.43 (−5.00, − 3.86)	−2.16 (−2.63, − 1.68)	3.21 (2.46,3.96)	−2.15 (−2.74, − 1.56)
College + /NonWhite/Rural	0.14 (−1.01,1.29)	−1.23 (−2.52,0.06)	2.80 (1.81,3.80)	7.22 (5.62,8.83)	1.70 (0.49,2.91)
College + /Nonwhite/Urban	−0.85 (−1.44, − 0.27)	−2.55 (−3.14, − 1.96)	2.72 (2.22,3.21)	7.68 (6.88,8.47)	1.74 (1.12,2.36)
College + /White/Rural	−1.82 (−2.44, − 1.21)	−0.92 (−1.55, − 0.30)	0.64 (0.11,1.16)	8.22 (7.38,9.06)	−0.82 (−1.50, − 0.15)
College + /White/Urban	−2.78 (−3.33, − 2.22)	−3.19 (−3.76, − 2.61)	0.76 (0.28,1.23)	9.36 (8.61,10.11)	−0.11 (−0.70,0.49)
p-value*	<0.001	<0.001	<0.001	<0.001	<0.001

* P-value is an overall Wald test that all interaction terms are jointly 0.

Compared with the group that identified as non-White, had less than a college degree, and lived in a rural area, groups that achieved a college degree were associated with approximately twice the odds of thriving in age- and sex-adjusted models (Table 4, Model 2). After adjusting for all covariates, including income, the group with the highest odds of thriving and the highest WBI score was the group identifying as non-White, with a college degree or higher, living in rural areas (Thriving OR: 1.54 (1.34,1.76); WBI b = 1.27 (0.41,2.14)) (Table 4, Model 4). The higher mean WBI score of this group was achieved through higher Financial (b = 7.22 (5.62,8.83)), Physical (b = 2.80 (1.81,3.80)), and Social (b = 1.70 (0.49,2.91)) well-being elements compared with the reference race-education-urban subgroup (Table 5). The subgroup with the lowest odds of thriving in fully adjusted models were respondents who identified as White, had less than a college degree, and were living in rural areas (OR=0.84 (0.79,0.90)) (Table 4, Model 4). The subgroup with the lowest WBI composite identified as White, had less than a college degree, and lived in urban areas (b = −2.74 (−3.16,-2.33)), which was driven primarily by relatively lower scores in the Community (b = −4.43 (−5.00,-3.86)) and Career (b = −3.90 (−4.45,-3.35)) elements (Table 5).

## Discussion

Findings from this study reveal complex relationships among sociodemographic characteristics and well-being that are reflective of longstanding structural racism that have limited opportunities for educational attainment, income, and location of residence based on one’s race/ethnicity. Though higher levels of educational achievement were consistently associated with higher odds of thriving and higher levels of well-being, its impact varied among race/ethnic subgroups. After adjusting for education and rural/urban residence, we revealed differences in odds of thriving and levels of well-being across racial/ethnic subgroups that varied in direction and strength depending upon additional adjustments for age, sex, marital status, household size, and income. Indeed, differences in well-being, as measured by the WBI, were driven primarily by differences in the Financial, Physical, and Career well-being elements. When assessing well-being across combination groups of race/ethnicity, educational attainment, and urban/rural residence, we found that the group who self-identified as non-White, with at least a college degree, and living in rural areas had the highest odds of thriving and reported the highest well-being.

Our findings suggest that structurally racist policies that have limited financial opportunity among non-White individuals compared with White individuals in the US with the same educational attainment for decades has had deleterious effects on their well-being. This is aligned with Keyes’ (2009) report using data from a nationally representative sample of middle-aged adults that Black individuals had higher rates of flourishing than White individuals after controlling for education and income—and this was higher after controlling for perceived discrimination [15]. The fact that in our study, the relative associations between Black and White race with measures of well-being changed in direction before and after adjustment for income and age has important implications for understanding the effects of structural racism. After adjusting for education and urbanicity, we found that participants who identified as Black had higher odds of thriving but lower levels of well-being. The financial return on investment is different for Black and White individuals, which manifests in a lower level of financial well-being among Blacks at a given education level. However, after further adjustment for income, Black participants of the same age as their White counterparts reported higher well-being. Our findings suggest that mitigating racial inequities in access to financial opportunity may be a mechanism to improve well-being.

Our study further highlights the complexity of relationships and interactions between race/ethnicity, education level, and rural or urban living environments. Most prior reports compare well-being between urban and rural residents, but we further assessed well-being among White and non-White residents with more or less than a college degree within these residential areas. The college-educated, non-White individuals living in rural areas that reported the highest rates of thriving and well-being comprised a very small portion of our sample, approximately 1500 participants (0.3%). However, this group was from all states and was evenly distributed among the non-White race/ethnicity categories. It is possible that these individuals report higher well-being in part because they are relatively better off than their parents and in part because they are relatively better off than others in their community. It is plausible that this subgroup of college-educated, non-White individuals were doing better than their parents, which may foster higher levels of optimism and their level of satisfaction with daily activities. It is also possible that this group, due to higher levels of optimism over the life course, invested more in their education and were more likely to succeed [16,17]. In addition, because this subgroup had higher education while living in rural areas with low cost of living, it is likely that they have higher relative income than most others in their community, supporting their experience of higher well-being [18,19]. This theory is supported by this groups’ higher relative scores in the Financial element.

Of note, these college-educated, non-White individuals living in rural and urban areas also reported better Physical well-being, which includes self-reported overall health and engagement in healthy behaviors, each of which have been linked to lower morbidity and mortality [20,21]. In this, our findings may at first appear to contradict what we might expect based on disparities in health outcomes well-described for some non-White groups and for rural populations [22–24]. For example, prior studies have shown that Black Americans have worse health outcomes than White Americans at all levels of income [25]. Importantly though, most of these prior analyses grouped all Black Americans together, including those living in urban and rural areas and with all levels of education. Though majority of this literature limits comparisons to people identifying as Black to those identifying as White, a recent study using data from the Multi-Ethnic Study of Atherosclerosis reports lower hazard of mortality from cardiovascular disease among Chinese and Hispanic participants compared with White participants [26]. We combined participants from these other race/ethnic groups together with Black participants, so we cannot determine whether a certain race/ethnic group drove the observed association. As an additional note, these prior studies did not explore whether well-being influenced associations with health outcomes. Our analyses may reveal weaknesses and some sources of residual confounding among prior studies, thereby highlighting areas for future research.

Our findings have notable public health implications. Assessment of well-being of these subgroups provides complementary and additional insights into the drivers of physical and mental health outcomes [27]. Well-being, an outcome with high intrinsic value, is also important to health outcomes of significance, such as longevity [28]. Efforts to increase length of life and mitigate disparities in life expectancy, though numerous, are unlikely to succeed if they do not also address well-being, as well-being is both a determinant and an outcome of health – that is, health contributes to well-being, and well-being contributes to health [29]. Perceived overall health alone is alone a strong predictor of mortality [20], and other positive psychosocial constructs related to well-being such as overall life satisfaction, social support, optimism, and emotion regulation have been linked prospectively with better health outcomes [16,30–33]. In addition, a recent report described low-income Americans experiencing the greatest race-based disparities in optimism: low-income Black Americans were most optimistic and low-income White Americans least optimistic, perhaps accounting for opposing trends in life expectancy gains among these two groups prior to the COVID-19 pandemic [34]. As such, it is plausible that identifying sociodemographic differences in a holistic measure of well-being may offer novel targets to address disparities in clinical health outcomes by strengthening positive elements of well-being rather than mitigating negative attributes associated with health outcomes [35].

Our study has several limitations to consider when interpreting results. First, because these data are collected by telephone survey, our results may be subject to response bias. The Gallup National Health and Well-Being survey has a twelve percent overall response rate. However, data from their sample has been successfully validated against other national surveys, such as the Behavioral Risk Factor Surveillance System [4]. Second, when we collapsed sociodemographic categories into dichotomous groups for the analyses on interactions, we lost the ability to potentially find differences among more granular subgroups. For example, we were unable to elicit any potential differences on returns on education in terms of well-being between Black, Hispanic, and Asian subgroups living in urban or rural environments. Future work should disentangle these effects. Third, we performed analyses using unweighted estimates of data collected using a sampling method designed to produce weighted population-based estimates. As such, our sample was more educated, had more respondents identifying as White, and fewer rural residents compared with US population averages. However, while this means our estimates of effect may be biased, findings of association remain valid because we are not extrapolating them to the population level. It is, however, possible that non-White, rural-dwelling respondents were more likely to experience higher well-being than non-White, rural-dwelling non-respondents. Finally, the years during which our data were collected (2014–2016) may have influenced respondents’ sense of optimism and well-being. Life evaluation can be shaped by the elected U.S. President at that time, and the data used in our analyses were collected during the second half of the Obama administration. While Black Americans’ life evaluation increased during Obama’s first term, it returned to typical levels in the second term [36,37]. Additionally, these years preceded the rise in well-being among residents of rural areas during the initial years of the Trump administration. As such, the data collected during 2014–2016 were not strongly influenced by politics at that time. Of note, our findings are also consistent with a multi-city sample of US community dwelling residents sampled during Winter 2016–2017 that reported higher odds of self-reported thriving among Black, Hispanic, and Asian populations compared with Whites, further suggesting that timing of our study may not have influenced results [38].

## Conclusion

Our findings reveal complex differences in well-being measures across race/ethnic subgroups in the US after controlling for education, which is the strongest predictor of thriving and level of well-being in the US. Several findings suggest that historical and current structural or systemic factors contribute to the observed disparities in well-being outcomes. The study also shows the importance of urban versus rural place of residence. These findings should prompt not only further study to understand more deeply and track these inequities but also community and societal level action to improve well-being and eliminate related inequities.
