# Supplementary material for: Describing well-being in the US by race/ethnicity, education level, and urbanicity: A cross-sectional study using the Gallup National Health and Well Being Index
Source: PLoS One. 2026 Sep 16;21(9):e0353745. doi: 10.1371/journal.pone.0353745 (PMC13580990; doi:10.1371/journal.pone.0353745)
Supplement: Peer review history

# Peer review: Decision Letter 0

**DOI:** 10.1371/journal.pone.0353745.r001

**Authors:** Angelina Wilson Fadiji

31 Mar 2026

Dear Dr. Roy,

Thank you for submitting your manuscript to PLOS ONE. After careful consideration, we feel that it has merit but does not fully meet PLOS ONE’s publication criteria as it currently stands. Therefore, we invite you to submit a revised version of the manuscript that addresses the points raised during the review process.

==============================

Please submit your revised manuscript by May 15 2026 11:59PM. If you will need more time than this to complete your revisions, please reply to this message or contact the journal office at plosone@plos.org. When you're ready to submit your revision, log on to https://www.editorialmanager.com/pone/ and select the 'Submissions Needing Revision' folder to locate your manuscript file.

- A letter that responds to each point raised by the academic editor and reviewer(s). You should upload this letter as a separate file labeled 'Response to Reviewers'.
- A marked-up copy of your manuscript that highlights changes made to the original version. You should upload this as a separate file labeled 'Revised Manuscript with Track Changes'.
- An unmarked version of your revised paper without tracked changes. You should upload this as a separate file labeled 'Manuscript'.

If you would like to make changes to your financial disclosure, please include your updated statement in your cover letter. Guidelines for resubmitting your figure files are available below the reviewer comments at the end of this letter.

If applicable, we recommend that you deposit your laboratory protocols in protocols.io to enhance the reproducibility of your results. Protocols.io assigns your protocol its own identifier (DOI) so that it can be cited independently in the future. For instructions see: https://journals.plos.org/plosone/s/submission-guidelines#loc-laboratory-protocols. Additionally, PLOS ONE offers an option for publishing peer-reviewed Lab Protocol articles, which describe protocols hosted on protocols.io. Read more information on sharing protocols at https://plos.org/protocols?utm_medium=editorial-email&utm_source=authorletters&utm_campaign=protocols.

As the corresponding author, your ORCID iD is verified in the submission system and will appear in the published article. PLOS supports the use of ORCID, and we encourage all coauthors to register for an ORCID iD and use it as well. Please encourage your coauthors to verify their ORCID iD within the submission system before final acceptance, as unverified ORCID iDs will not appear in the published article. *Only*  the individual author can complete the verification step; PLOS staff *cannot*  verify ORCID iDs on behalf of authors.

We look forward to receiving your revised manuscript.

Kind regards,

Angelina Wilson Fadiji, PhD

Academic Editor

PLOS One

Journal Requirements:

If the reviewer comments include a recommendation to cite specific previously published works, please review and evaluate these publications to determine whether they are relevant and should be cited. There is no requirement to cite these works unless the editor has indicated otherwise.

Please review your reference list to ensure that it is complete and correct. If you have cited papers that have been retracted, please include the rationale for doing so in the manuscript text, or remove these references and replace them with relevant current references. Any changes to the reference list should be mentioned in the rebuttal letter that accompanies your revised manuscript. If you need to cite a retracted article, indicate the article’s retracted status in the References list and also include a citation and full reference for the retraction notice.

[Note: HTML markup is below. Please do not edit.]

Reviewers' comments:

Reviewer's Responses to Questions

**Comments to the Author**

1. Is the manuscript technically sound, and do the data support the conclusions?

Reviewer #1: Yes

Reviewer #2: Yes

**********

2. Has the statistical analysis been performed appropriately and rigorously?

Reviewer #1: Yes

Reviewer #2: Yes

**********

3. Have the authors made all data underlying the findings in their manuscript fully available?

The PLOS Data policy

Reviewer #1: Yes

Reviewer #2: No

**********

4. Is the manuscript presented in an intelligible fashion and written in standard English?

Reviewer #1: Yes

Reviewer #2: Yes

**********

Reviewer #1: Study design matches the aims. Statistical analysis seems adequate. All table seems valid.

The study findings suggest complex differences in well-being measures across race/ethnic subgroups' in the US after controlling for education, which is the strongest predictor of thriving and level of well-being in the US.

Reviewer #2: I really found this manuscript; on describing well-being in the US by race/ethnicity, educational level, and urbanization; a strong one and an important contribution to existing literature in the area. The methodological approach and the comprehensive way through which the authors explained their data analysis and results is a notable strength of the manuscript. I found that the manuscript could benefit from the inclusion of greater conceptual detail in the Introduction regarding what the authors have named "multidimensional assessment of well-being and its elements". Although some operational descriptions are given in the methods section, the reference to the elements cannot be left undescribed at this introduction point. Given the findings of the study, which the authors acknowledge to being different given previous literature, particularly on race; it may be beneficial to also reference a study by Keyes (2009), in which he referred to such findings as a race paradox: Keyes, C. (2009). The Black-White paradox in health: Flourishing in the face of social inequality and discrimination. Journal of Personality, 77(6), 1677-1705.

**********

what does this mean?). If published, this will include your full peer review and any attached files.

If you choose “no”, your identity will remain anonymous but your review may still be made public.

**Do you want your identity to be public for this peer review?**  For information about this choice, including consent withdrawal, please see our Privacy Policy

Reviewer #1: **Yes:**  Senthamizhchelvan Srinivasan

Reviewer #2: **Yes:**  Itumeleng P Khumalo

**********

[NOTE: If reviewer comments were submitted as an attachment file, they will be attached to this email and accessible via the submission site. Please log into your account, locate the manuscript record, and check for the action link "View Attachments". If this link does not appear, there are no attachment files.]

To ensure your figures meet our technical requirements, please review our figure guidelines: https://journals.plos.org/plosone/s/figures

You may also use PLOS’s free figure tool, NAAS, to help you prepare publication quality figures: https://journals.plos.org/plosone/s/figures#loc-tools-for-figure-preparation.

NAAS will assess whether your figures meet our technical requirements by comparing each figure against our figure specifications.

# Author comment: Author response to Decision Letter 1

**DOI:** 10.1371/journal.pone.0353745.r002

20 May 2026

May 14, 2026

Angela Wilson Fajidi

Academic Editor, PLOS One

Dear Dr. Fadiji,

I would like to thank you and the reviewers for your careful critique of our manuscript, “Describing well-being in the US by race/ethnicity, education level, and urbanicity: a cross-sectional study using the Gallup National Health and Well Being Index.” (PONE-D-25-15075). My co-authors and I appreciate the opportunity to revise and resubmit. Below are the reviewer’s comments and our responses:

Reviewer #1: Study design matches the aims. Statistical analysis seems adequate. All table seems valid.

The study findings suggest complex differences in well-being measures across race/ethnic subgroups' in the US after controlling for education, which is the strongest predictor of thriving and level of well-being in the US.

We thank the reviewer for their endorsement of the manuscript’s methods and findings.

Reviewer #2: I really found this manuscript; on describing well-being in the US by race/ethnicity, educational level, and urbanization; a strong one and an important contribution to existing literature in the area. The methodological approach and the comprehensive way through which the authors explained their data analysis and results is a notable strength of the manuscript.

We thank the reviewer for their compliments on the strength of the contribution of this paper and rigor of the methodologic approach.

I found that the manuscript could benefit from the inclusion of greater conceptual detail in the Introduction regarding what the authors have named "multidimensional assessment of well-being and its elements". Although some operational descriptions are given in the methods section, the reference to the elements cannot be left undescribed at this introduction point.

We have added the reference to the multidimensional assessment of well-being and have added the names of the elements in the introduction (lines 88-92).

Given the findings of the study, which the authors acknowledge to being different given previous literature, particularly on race; it may be beneficial to also reference a study by Keyes (2009), in which he referred to such findings as a race paradox: Keyes, C. (2009). The Black-White paradox in health: Flourishing in the face of social inequality and discrimination. Journal of Personality, 77(6), 1677-1705.

Thank you for suggesting this reference. We are aware of Keyes’ seminal work, and we were remiss not to include it in the original submission. A description this study’s aligment with our findings along with the reference has been added to the discussion (lines 357-61).

Please do not hesitate to let me know if any further changes are required. Again, we thank you for your time and for considering our manuscript for publication in PLOS One.

Sincerely,

Brita Roy

# Editor report: Decision Letter 1

**DOI:** 10.1371/journal.pone.0353745.r003

**Authors:** Angelina Wilson Fadiji

29 Jun 2026

Describing well-being in the US by race/ethnicity, education level, and urbanicity: a cross-sectional study using the Gallup National Health and Well Being Index

PONE-D-25-15075R1

Dear Dr. Roy

We’re pleased to inform you that your manuscript has been judged scientifically suitable for publication and will be formally accepted for publication once it meets all outstanding technical requirements.

Within one week, you’ll receive an e-mail detailing the required amendments. When these have been addressed, you’ll receive a formal acceptance letter and your manuscript will be scheduled for publication.

An invoice will be generated when your article is formally accepted. Please note, if your institution has a publishing partnership with PLOS and your article meets the relevant criteria, all or part of your publication costs will be covered. Please make sure your user information is up-to-date by logging into Editorial Manager at Editorial Manager® and clicking the ‘Update My Information' link at the top of the page. For questions related to billing, please contact billing support.

If your institution or institutions have a press office, please notify them about your upcoming paper to help maximize its impact. If they’ll be preparing press materials, please inform our press team as soon as possible -- no later than 48 hours after receiving the formal acceptance. Your manuscript will remain under strict press embargo until 2 pm Eastern Time on the date of publication. For more information, please contact onepress@plos.org.

Kind regards,

Angelina Wilson Fadiji, PhD

Academic Editor

PLOS One

Additional Editor Comments (optional):

Reviewers' comments:

# Editor report: Acceptance letter

**DOI:** 10.1371/journal.pone.0353745.r004

**Authors:** Angelina Wilson Fadiji

PONE-D-25-15075R1

PLOS One

Dear Dr. Roy,

I'm pleased to inform you that your manuscript has been deemed suitable for publication in PLOS One. Congratulations! Your manuscript is now being handed over to our production team.

At this stage, our production department will prepare your paper for publication. This includes ensuring the following:

* All references, tables, and figures are properly cited

* All relevant supporting information is included in the manuscript submission,

* There are no issues that prevent the paper from being properly typeset

You will receive further instructions from the production team, including instructions on how to review your proof when it is ready. Please keep in mind that we are working through a large volume of accepted articles, so please give us a few days to review your paper and let you know the next and final steps.

Lastly, if your institution or institutions have a press office, please let them know about your upcoming paper now to help maximize its impact. If they'll be preparing press materials, please inform our press team within the next 48 hours. Your manuscript will remain under strict press embargo until 2 pm Eastern Time on the date of publication. For more information, please contact onepress@plos.org.

You will receive an invoice from PLOS for your publication fee after your manuscript has reached the completed accept phase. If you receive an email requesting payment before acceptance or for any other service, this may be a phishing scheme. Learn how to identify phishing emails and protect your accounts at https://explore.plos.org/phishing.

If we can help with anything else, please email us at customercare@plos.org.

Thank you for submitting your work to PLOS One and supporting open access.

Kind regards,

PLOS One Editorial Office Staff

on behalf of

Dr. Angelina Wilson Fadiji

Academic Editor

PLOS One
